# The Prostaglandin EP3 Receptor Is an Independent Negative Prognostic Factor for Cervical Cancer Patients

**DOI:** 10.3390/ijms18071571

**Published:** 2017-07-19

**Authors:** Helene Heidegger, Sebastian Dietlmeier, Yao Ye, Christina Kuhn, Aurelia Vattai, Caroline Aberl, Udo Jeschke, Sven Mahner, Bernd Kost

**Affiliations:** 1 Department of Obstetrics and Gynecology, Ludwig-Maximilians-University of Munich, Campus Innenstadt, Maistraße 11, 80337 Munich, Germany; helene.heidegger@med.uni-muenchen.de (H.H.); sebastian.dietlmeier@med.uni-muenchen.de (S.D.); Yao.Ye@med.uni-muenchen.de (Y.Y.); Christina.kuhn@med.uni-muenchen.de (C.K.); Aurelia.vattai@med.uni-muenchen.de (A.V.); caroline.aberl@med.uni-muenchen.de (C.A.); sven.mahner@med.uni-muenchen.de (S.M.); bernd.kost@med.uni-muenchen.de (B.K.); 2 Department of Obstetrics and Gynecology, Ludwig-Maximilians-University of Munich, Campus Großhadern, Marchionistraße 15, 81377 Munich, Germany

**Keywords:** cervical cancer, squamous cell carcinoma, adenocarcinoma, EP3 receptor, overall survival, prognostic factor, cox regression

## Abstract

We know that one of the main risk factors for cervical cancer is an infection with high-risk human papillomavirus (HR-HPV). Prostaglandins and their receptors are very important for the tumour growth and tumour-associated angiogenesis. Little is known about the expression of the Prostaglandin E receptor type 3 (EP3) or the Prostaglandin (PG)E_2_-EP3 signalling in cervical cancer, so the aim of the study was to analyse the expression of the EP3 receptor in cervical cancer and find prognostic factors in relation to survival; EP3 immunohistological staining of 250 cervical cancer slides was performed and analysed with a semi-quantitative score. The statistical evaluation was performed with Statistical Package for the Social Sciences (SPSS) to evaluate the staining results and the survival analyses of the cervical cancer cases. A significant difference was observed in EP3 expression in Fédération Internationale de Gynécologie et d’Obstétrique (FIGO) stadium I versus FIGO stadium II–IV cases. High expression of EP3 (IRS ≥ 1.5) in cervical cancer patients was correlated with poor prognosis in overall survival rates. Survival in adenocarcinoma (AC) of the cervix was lower than in squamous cell carcinoma (SCC). Cox regression analysis shows that EP3 is an independent prognosticator. In this study we could show that the membrane-bound prostaglandin receptor EP3 is an independent prognosticator for cervical cancer patient survival. Targeting the EP3 receptor seems to be an interesting candidate for endocrine therapy. Therefore, more research is needed on the influence of the receptor system and its influence on cervical cancer growth.

## 1. Introduction

Approximately half a million women are diagnosed annually with invasive cervical cancer worldwide. In the year 2012 we had about 530,000 new cases, which is about 8% of all female cancer deaths [1]. The infection with genital human papillomavirus (HPV) is one of the most common sexually-transmitted infections worldwide [2]. We know that one of the main risk factors for cervical cancer is an infection with high-risk human papillomavirus (HR-HPV). Especially HPV-16 and HPV-18 subtypes cause nearly 70% of all cases of cervical cancer [3]. The most common HPV subtypes in woman with normal cytological findings are HPV-16, HPV-18, HPV-52, HPV-31, and HPV-58 [2].

Prostanoids are metabolites of arachidonic acid synthesized by cyclooxygenase-1 (COX-1) and cyclooxygenase-2 (COX-2) [4]. The prostaglandin (PG) D_2_, PGE_2_, PGF_2α_, PGI_2_, and the thromboxane A_2_ are found in most tissues and organs. They are produced by almost all nucleated cells and act as autocrine and paracrine lipid mediators [5]. Each prostaglandin has, as a ligand, its own receptor. The receptor for the PGE_2_, named EP receptor, has four subtypes (EP1, EP2, EP3, EP4). The receptors are G protein-coupled receptors with seven transmembrane domains [6]. The prostaglandins play an important role in the induction of fever, pain, infection, immunity, and the stimulation of the hypothalamic-pituitary-adrenal axis [7]. Some of the prostaglandins are implicated in many aspects of reproductive functions. In addition the PG play an important role in vascular homeostasis, like inducing hypertension, thrombosis, and haemostasis [6].

The prostaglandins and their receptors are very important for tumour growth and tumour-associated angiogenesis. However, the identity of the responsible prostaglandins and the prostaglandin receptors is at the moment unknown [8]. Amano et al. characterized the role of PG-signalling in tumour-associated angiogenesis and tumour progression in a mouse model and declare that the PGE_2_-EP3 signalling is critical for tumour-associated angiogenesis and tumour growth [8]. Recent studies suggest that many tumours are regulated by COX enzyme products [9]. COX-2 is upregulated in numerous cancers like pancreas, lung, bladder, colon, and prostate [10].

The EP3 receptor subtype is very special among the EP receptors, because in that there are multiple isoforms generated through mRNA splicing. So various splicing variants have been identified [11,12,13]. The different isoforms differ in the C-terminus and through different signal transduction pathways [13]. Regarding the EP3 isoforms and their effects, little is known and their different physiological roles remain unknown [12].

Little is known about the expression of the EP3 receptor or the PGE_2_-EP3 signalling in cervical cancer. A few studies suggest the overexpression of COX-2 in cervical cancer [14]. However, the mechanism of the upregulation of COX-2 in cervical cancer remains unknown [15].

The aim of this study was a systematic analysis of the expression of the EP3 receptor in human squamous cell carcinomas and adenocarcinomas of the cervix. In addition, we want to investigate if there exists some prognostic factors in relation to survival. A selective EP3 antagonist may exhibit a chemoprotective effect and, in the future, it could become a new important tool for cancer therapy [8].

## 2. Results

### 2.1. Positive Control of EP3 Staining

**Figure 1:**
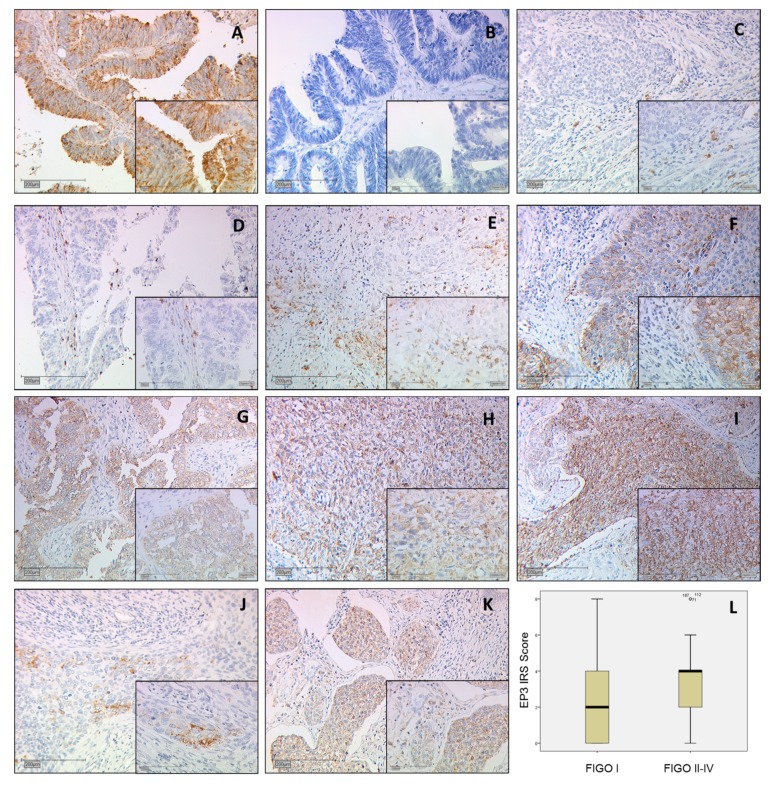
All images are at 10× magnification with an insert at 25× magnification. (**A**) Positive control of ovarian cancer metastasis in the colon shows cystoplasmatic and membrane-associated staining; (**B**) Negative control of ovarian cancer metastasis in the colon; (**C**) Squamous cell carcinoma Immunoreactive score (IRS) 1; (**D**) Adenocarcinoma IRS 1; (**E**) Squamous cell carcinoma IRS 4; (**F**) Adenocarcinoma IRS 4; (**G**) Adenocarcinoma carcinoma IRS 8; (**H**) Squamous cell carcinoma IRS 8, (**I**) Squamous cell carcinoma IRS 9; (**J**) Prostaglandin E receptor type 3 (EP3) staining of an Fédération Internationale de Gynécologie et d’Obstétrique (FIGO) Ib diagnosed IRS 2 stained squamous cell carcinoma; (**K**) EP3 staining of an FIGO 4 diagnosed IRS 4 stained squamous cell carcinoma; (**L**) Boxplot of FIGO I and FIGO II–IV cases with median IRS.

Paraffin-embedded sections of ovarian carcinoma metastasis in the colon were used to control the quality of the EP3 staining (Figure 1A,B). The anti-PTGER3 antibody binding site, the first cytoplasmatic domain with the amino acid sequence: RRESKRKKSFLLC position 79−91 is present in all EP3 isoforms 1–12 and was picked after researching the Human Protein Atlas.

### 2.2. EP3 Staining in Cervical Carcinoma

The intensity of the expression was evaluated by the immunoreactive score (IRS) using a Leitz (Wetzlar, Germany) microscope, and is well-established and applied in numerous other studies. In brief, this semi-quantitative score multiplies the intensity of the staining (0 = not stained; 1 = low intensity; 2 = moderate intensity; 3 = high intensity) and the percentage of stained cells (0 = 0%; 1 = 1–10%; 2 = 11–50%; 3 = 51–80%; 4 ≥ 80%). Finally, we distinguished between 0 = no expression and 12 = very high expression of EP3 [16]. Two independent observers were blinded and evaluated the intensity and distribution pattern of the immunochemical staining reaction. The two observers differed in eight cases (*n* = 3.2%) of the evaluation. These cases were re-evaluated together and both observers came to the same result. The concordance before the re-evaluation was 96.8%.

A total of 77.2% of all cervical cancer specimens showed cytosolic expression of EP3. The IRS was 2.75 in 76% of the samples, compared to cases that did not express EP3 (18.0%) at all. Compared to 21.1% with low expression (IRS < 1.5), an enhanced staining (IRS ≥ 1.5) was detected in 78.9% of the samples. The cut off of IRS 1.5 was obtained through receiver operator curve (ROC) analysis. We found significant positive correlation using Spearman’s test between EP3 IRS staining and tumor size (pT) (*p* = 0.018; Rho = 0.154) and FIGO stadium (*p* = 0.040; Rho = 0.133).

We separated two groups regarding invasiveness: FIGO stadium patients with the diagnosis of FIGO I, IA, IB (Figure 1J) which have a limited tumour in the cervical part of the uterus and the second group with FIGO II, III, IV (Figure 1K) stadium. The result was that the first group of 57 cases had a median EP3 IRS score of 2 and the second group of 91 cases showed a median EP3 IRS score of 4 with a significance of *p* = 0.012 (Figure 1L).

### 2.3. Correlation Analysis between Prostaglandin E Receptor Type 3 (EP3) and Fédération Internationale de Gynécologie et d’Obstétrique (FIGO) Classification

We examined the correlation between EP3 and several clinic pathological parameters, such as grading, histology, size of the primary tumour (T-status), nearby lymph nodes (N-status), and FIGO-classification by noticing the distribution of these parameters in our study group. In addition, a significant difference was observed in EP3 expression in FIGO stadium I cases versus FIGO stadium II–IV cases (Table 1).

### 2.4. Role of EP3 for Overall Survival

**Figure 2:**
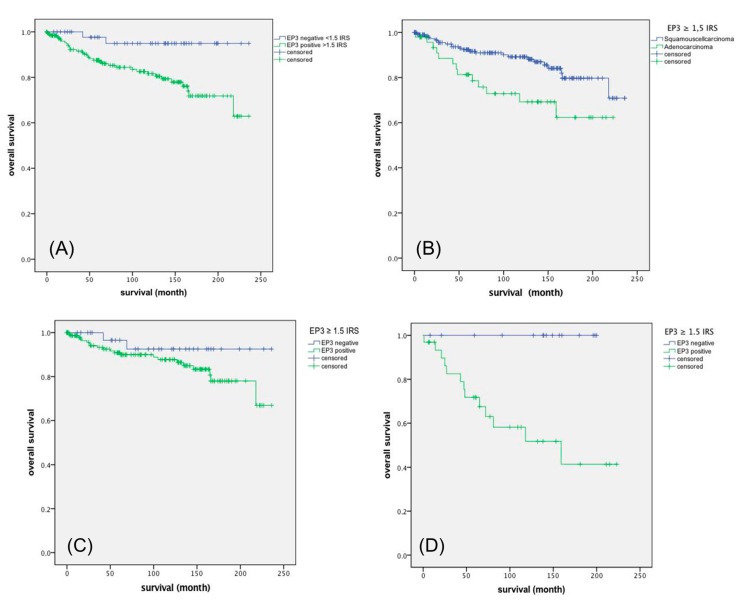
Kaplan-Meier curves: (**A**) EP3 survival function of all cervical cancer carcinoma *p* = 0.012; (**B**) EP3 survival function of all cervical squamous-versus adenocarcinoma *p* = 0.009; (**C**) EP3 survival function of cervical squamous cell carcinoma diagnosed patients *p* = 0.003; (**D**) EP3 survival function of cervical adenocarcinomas patients *p* = 0.003.

Enhanced EP3 expression (IRS ≥ 1.5, obtained by ROC-analysis) was associated with shorter survival time after diagnosis. As shown in the Kaplan-Meier curve (Figure 2A), high expression of EP3 (IRS ≥ 1.5) in cervical cancer patients was correlated with poor prognosis in overall survival rates (*p* = 0.012).

### 2.5. Survival Function of Squamous Cell Carcinoma Versus Adenocarcinoma

Additionally, we compared the cumulative survival of all EP3-positive (IRS ≥ 1.5) squamous cell carcinomas versus adenocarcinomas. The Kaplan-Meier curve (Figure 2B) shows, as expected, that adenocarcinoma patients have a poor survival time after diagnosis *p* = 0.009 [17].

### 2.6. EP3 Staining of Squamous Cell Carcinoma Versus Adenocarcinoma

In addition, we performed a Kaplan-Meier test for EP3 positive (IRS ≥ 1.5) squamous cell carcinoma and adenocarcinomas versus their EP3 negative ones and were able to show that EP3 expression in squamous carcinoma patients is significant with poor survival (*p* = 0.003; Figure 2C). Overall survival in cervical adenocarcinomas indicates that none of the EP3 negative patients in our collective died (Figure 2D).

### 2.7. Cox Regression of EP3 Immunoreactive Score (IRS) with Clinic Pathological Variables

The additionally performed multivariate cox-regression tested which histopathological parameter were independent prognosticators for survival in our study group.

For overall survival the histological subtype (*p* = 0.002), lymph node metastasis (pN)-status (*p* = 0.025) and tumor size (pT)-status (*p* = 0.001) were independent prognosticators (Table 2).

## 3. Discussion

In recent years, attention has been focused on understanding the role of inflammation in tumour biology. It is known that COX-2 plays an important role for the induction of inflammation either individually or through sustained production of PGE_2_ [18]. The overexpression of COX-2 is reported in numerous human malignancies including colon, breast, lung, and prostate [19]. It is even reported that COX-2 is overexpressed in HPV-related diseases, like cervical cancer [20].

In our investigation we examined the expression of EP3 receptor in cervical cancer (adenocarcinoma and squamous cell carcinoma), the EP3 receptor as an independent marker and tried to find prognostic factors in relation to survival.

Within this study, we showed that the immunohistochemical evaluation of EP3 receptor staining was correlated with high FIGO-classification in cervical cancer. This is in line with prognostic implications of higher EP3 receptor expression and higher FIGO-classification, which are both associated with poorer survival. We demonstrated that an increased EP3 receptor expression correlates with a negative outcome of overall survival of cervical carcinoma patients.

Further studies suggest that tumour histology has an important impact on survival for women with cervical cancer and, additionally, a poorer survival in patients with cervical adenocarcinoma [21]. We find the same results but, additionally, we could demonstrate that patients with adenocarcinoma and an IRS less than 1.5 had a very good overall survival rate. It is useful to distinguish between patients with AC and patients with AC and a high expression of EP3 receptor too, because the latter had a significantly worse outcome regarding survival. Thus, targeting the EP3 receptor, diagnostically, generally seems possible. On the other hand a new study from 2017 suggest that there was no significant difference in survival when patients were compared by cell type, so the prognosis of adenocarcinoma is controversially discussed in the literature and further studies are required [22].

The frequency of cervical adenocarcinoma is variable, but a prevalence between 15% and 25% is reported in the current literature [23]. Although the AC is less frequent than the SCC, we think that an immunohistochemical evaluation of the EP3 receptor could be an interesting tool for the clinical routine in the future.

In our study next to the EP3 receptor we found the T-status, the histology and the N-status to be an independent marker of overall survival. To our knowledge, this is the first time that associations of EP3 receptor with other biological characteristics of cervical cancer and the effect of EP3 on survival of cervical cancer patients have been analysed. We could not find another report describing EP3 as an independent prognosticator for long time survival in cervical cancer patients. Other independent markers for overall survival in patients with cervical cancer have also been investigated by Beyer et al. [16]. They found the histone H3 acetyl K9 to be an independent marker of overall survival. Chen et al. supposed that cervical carcinoma high-expressed long non coding RNA 1 (lncRNA-CCHE1) is an independent poor prognostic biomarker [24].

The role of EP3 and cancer in other studies show various effects. Some studies demonstrate an indirect pro-tumorigenic effect of EP3 receptor expression in various kinds of cancer, which was similar to our data. Miyata et al. have shown that the density of EP3 receptor positive stromal cells is associated with cancer cell progression and malignant potential, including angiogenesis and lymphangiogenesis [25]. The EP3 receptor has been shown to contribute to malignant aggressiveness, carcinogenesis and poor prognosis in several cancer types like lung adenocarcinoma and breast carcinoma [26]. On the contrary, other studies suggest an anti-tumorigenic effect of EP3 receptor expression. Shoji et al. show a colon tumour development in EP3 receptor knockout mice and suggest an important role of EP3 in suppression of cell growth [27]. Another study shows that an upregulation of EP3 expression in prostate cancer cells has preventive and anticancer effects [28,29].

Important to respect is the fact that we have different isoforms of the EP3 receptor. Many details of the EP3 receptor and its isoforms are uncovered and the data have a number of discrepancies, especially with regard to its effects [12]. The isoforms of the EP3 receptor may have different effects and physiological roles based on the tissue, in which they are expressed [12]. Thus, further studies are required to investigate the PGE_2_/EP3 isoforms for a better understanding of the physiological and pathophysiological effects.

## 4. Materials and Methods

### 4.1. Patients and Specimens

In this study, cervical cancer tissue samples of 250 patients who underwent surgery for cervical cancer from 1993 to 2002 at the Department of Gynecology and Obstetrics, Ludwig-Maximilians-University of Munich, Germany were used. The patient’s median age was 47 years (range 20–83 years), and overall median survival was 100 months. The distribution of clinic-pathological variables can be seen in Table 3. In our study patients with squamous cell carcinoma or adenocarcinoma of the cervix were included, other histological subtypes were excluded due to the low number. No pre-selection besides that took place. As a positive control for immunohistochemical staining, we utilized ovarian carcinoma metastasis of the colon tissue for EP3 which was received from the Department of Obstetrics and Gynecology of the Ludwig-Maximilians-University of Munich. The Munich Cancer Registry (MCR) provided clinical and follow-up data for statistical analyses and retrieved from medical records. All of this is supported by the Bavarian Cancer Registry act and results in a loss of 4.4% follow-up patients.

### 4.2. Ethics Approval

The initially collected cervical cancer specimens for histopathological diagnostics were no longer used for clinical tests. We recruited all patients for this survey out of this histopathological collective.

The data of the patients were totally anonymised. The authors were blinded for clinical information during statistical analyses, including survival time. The ethics committee of the Ludwig-Maximilians University approbated the ethical vote of this study. The Helsinki Declaration guidelines were respected (reference number 259-16, 13 June 2016).

### 4.3. Immunohistochemistry

The paraffin-embedded and formalin-fixed samples were cut (3 µm) from all specimens and mounted on positively charged glass slides. Stored at +20 °C before dewaxing for 20 min in xylol was performed. After washing the tissue in 100% ethanol, the endogenous peroxidase was blocked with 3% methanol/H_2_O_2_ for 20 min. The tumour slides were rehydrated in a descending alcohol series. To unmask the antigen after formalin-fixation-associated protein-agglomeration, the slides were warmed up in an airtight pot for 5 min at +100 °C, adding a trisodium citrate buffer solution (Merck 244 and Merck 6448) with pH = 6. After preparing the slides by washing them in distilled water and PBS-buffer the first step of the Polymer kit (ZytoChem Plus HRP Polymer System, Berlin, Germany) was applied for 5 min to avoid unspecific (hydrophobic) bindings. Incubation of the samples at +4 °C for 16 h with the EP3 primary antibody (anti-PTGER3 antibody polyclonal rabbit IgG; ABCAM ab189131) followed. After steps 2 and 3 of the polymer kit (Reagents 2 and 3), the substrate-staining with DAB (chromogen substrate kit, Dako, Munich, Germany) was performed for two and a half minutes, followed by the counterstaining by hemalaun colouring (2 min). The samples were finally dehydrogenated in an ascending alcohol series and covered.

## 5. Conclusions

In this study we showed that the immunohistochemical evaluation of the EP3 receptor expression is correlated to the FIGO classification, so we could demonstrate that an increased EP3 receptor expression correlates with a negative outcome of overall survival of cervical carcinoma patients.

In addition we found a different expression of EP3 in correlation to the histological subtype. Patients with AC and a high expression of EP3 receptor had a significant worse outcome regarding survival. Targeting the EP3 receptor diagnostically seems generally possible.

## Figures and Tables

**Table 1:** EP3 Immunoreactive score (IRS) staining results and correlation analysis, pN = lymph node stage, pT = tumour stage, FIGO = Fédération Internationale de Gynécologie et d’Obstétrique.

Variables	*p* (NPAR)	Correlation Coefficient
Histology	0.700	(−0.025)
pN	0.229	0.078
pT	0.018	0.154
FIGO	0.040	0.133

**Table 2:** Cox regression of clinic pathological variables regarding overall survival, pM = distant metastasis stage, IRS = Immunoreactive score, CI = confidence interval, Exp (B) = hazard ratio.

Variable	Significance	Hazard Ratio of Exp (B)	Lower 95% CI of Exp (B)	Upper 95% CI of Exp (B)	B
EP3 IRS	0.007	1.264	1.066	1.498	0.234
Histology	0.002	3.118	1.538	6.322	1.137
pT	0.001	1.32	1.115	1.562	0.277
pN	0.025	2.208	1.103	4.42	0.792
FIGO	0.398	0.971	0.905	1.04	−0.030
Grading	0.242	1.381	0.804	2.372	0.323
Age	0.136	1.021	0.993	1.05	0.021
pM	0.261	2.214	0.554	8.857	2.214

**Table 3:** Patient characteristics.

Item	Numbers/Total Numbers	Percentage
**Age**		
<49	139/250	55.6%
>49	111/250	44.4%
**Number of positive lymph nodes**		
0	151/250	60.4%
>1	97/250	38.8%
Not available	2/250	0.8%
**pT, Tumour size**		
pT1	110/250	44,0%
pT2/3/4	137/250	54.8%
Not available	3/250	1.2%
**FIGO**		
I	64/250	25.6%
II/III/IV	92/250	36.8%
Not available	94/250	37.6%
**Tumour grade**		
I	21/250	8.4%
II	143/250	57.2%
III	78/250	31.2%
Not available	8/250	3.2%
**Tumour subtype**		
Squamous	202/250	80.8%
Adenocarcinoma	48/250	19.2%
**Progression (over 235 months)**		
None	210/250	84,0%
At least one event	21/250	11.6%
Not available	11/250	4.4%
**Survival (over 235 months)**		
Right censured	190/250	76,0%
Died	49/250	19.6%
Not available	11/250	4.4%
